# Concise methods for the synthesis of chiral polyoxazolines and their application in asymmetric hydrosilylation

**DOI:** 10.3762/bjoc.6.29

**Published:** 2010-03-25

**Authors:** Wei Jie Li, Zun Le Xu, Sheng Xiang Qiu

**Affiliations:** 1 Program for Natural Product Chemical Biology & Drug Discovery, South China Botanical Garden, Chinese Academy of Sciences, 723 Xingke Road, Tianhe District, Guangzhou 510650, China; 2 Department of Applied Chemistry, School of Chemistry and Chemical Engineering, Sun Yat-Sen University, 135 Xingang West Road, Haizhou District, Guangzhou 510275, China

**Keywords:** aromatic ketone, hydrosilylation, polyoxazoline, rhodium, synthesis

## Abstract

Seven polyoxazoline ligands were synthesized in high yield in a one-pot reaction by heating polycarboxylic acids or their esters and chiral β-amino alcohols under reflux with concomitant removal of water or the alcohol produced in the reaction. The method is much simpler and more efficient in comparison to those methods reported in the literature.

The compounds were used as chiral ligands in the rhodium-catalyzed asymmetric hydrosilylation of aromatic ketones, and the effects of the linkers and the substituents present on the oxazoline rings on the yield and enantioselectivity investigated. Compound **2** was identified as the best ligand of this family for the hydrosilylation of aromatic ketones.

## Introduction

The design and development of effective chiral oxazoline ligands have played a significant role in the advancement of asymmetric catalysis and have attracted a great deal of attention. Various chiral oxazoline ligands have been developed and applied in many catalytic asymmetric reactions to prepare enantiomerically pure compounds; in particular, a range of mono- and bisoxazolines have been widely used as effective templates for metal-catalyzed asymmetric reactions over the last 30 years [1–13]. Previously, polyoxazoline ligands were reported to have good catalytic activities and high enantioselectivities in various asymmetric reactions [14–20]. For example, chiral 1,2,2-tris[2-(4-isopropyloxazolinyl)]propane was shown to lead to high enantioselectivities in the Cu(II)-catalyzed asymmetric Michael addition reaction between indole and alkylene malonates [19]. More recently, the complex from 2,2′,6,6′-tetrakis[(4*S*)-phenyloxazolin-2-yl]-biphenyl and Pd(II) was reported to show excellent catalytic activities and enantioselectivities in the Wacker-type cyclization of allylphenols with up to 99% ee [20]. Despite their great application potential, until now, only a few polyoxazoline ligands have been reported in the literature due to synthetic difficulties. In general, the syntheses of polyoxazolines from polycarboxylic acids or polycarboxylates and chiral amino alcohols are carried out via poly(β-hydroxyamide)s as intermediates, followed by cyclization to afford the target compounds. The methods require activating agents or cyclizing agents such as thionyl chloride, methanesulfonic chloride or PPh_3_ etc. [19–21], which result in more side reactions and low yields. Therefore, simpler and more efficient synthetic strategies are required for the preparation of polyoxazoline ligands.

**Figure 1:**
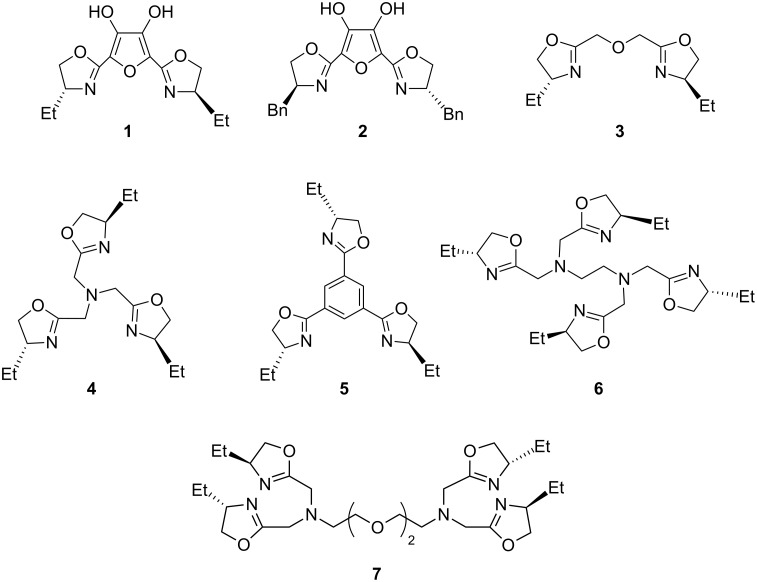
Chemical structures of polyoxazolines.

In the present study, we report the results of heating polycarboxylic acids or their esters with chiral β-amino alcohols under reflux conditions with the simultaneous removal of water or the alcohol produced in the reaction in a one-step process for the preparation of novel chiral polyoxazoline ligands (Figure 1). These processes are high yield reactions with simple workup procedures. Furthermore, we have also investigated the reactivities and resulting enantioselectivities of the newly synthesized ligands in the asymmetric hydrosilylation catalyzed by [Rh(COD)Cl]_2_.

## Results and Discussion

### Syntheses of polyoxazolines

The conventional methods for the preparation of polyoxazolines from polycarboxylic acids or their esters have been often carried out via poly(β-hydroxylamide)s intermediates; the latter were then cyclized in the presence of condensing agents such as thionyl chloride/NaOH, methanesulfonic chloride or PPh_3_ etc. to produce the desired compounds [19–21]. However, these methods are associated with side reactions and low yields. To address these issues, we have successfully developed facile procedures for the syntheses of polyoxazolines starting from polycarboxylic acids or their esters, such as 3,4-dihydroxyfuran-2,5-dicarboxylic acid (DFA) and its dimethyl ester (DDFA), diglycolic acid and its dimethyl ester, triglycine and its triethyl ester, 1,3,5-benzenetricarboxylic acid (BTA) and its trimethyl ester (TBTA), ethylenediaminetetraacetic acid (EDTA) and ethylene glycol-bis(2-aminoethylether)-*N*,*N*,*N*′,*N*′-tetraacetic acid (EGTA).

As the data in Table 1 indicates, DFA, diglycolic acid or their dimethyl esters, respectively, react with (*R*)-2-amino-1-butanol or L-phenylalaninol in toluene under reflux in 18–23 h with the elimination of water or methanol in a one-pot reaction, to give the bisoxazolines **1**–**3** in good yields (entries 1–6). However, when triglycine or its triethyl ester, BTA or TBTA, was heated with (*R*)-2-amino-1-butanol in toluene, good yields of the desired products were not obtained, even after 24 h. To resolve this problem, the toluene was removed and the resulting mixture stirred for 6–9 h at 125 °C to complete the reaction. By employing this modified procedure, the trioxazolines **4** or **5** were obtained in excellent yields (entries 7–10). Similarly, when a mixture of EDTA or EGTA and (*R*)- or (*S*)-2-amino-1-butanol in toluene was refluxed for 20 h, the tetraoxazolines **6** or **7** were obtained in poor yield. Again, after toluene was removed and the resulting mixtures stirred for 8 h at 135 °C, the desired tetraoxazolines were obtained in high yields (entries 11 and 12).

**Table 1:** The conditions and results of the reaction of polycarboxylic acids or their esters with chiral β-amino alcohols.

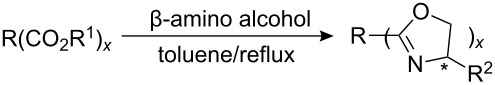							
**Entry**	**Reaction substrate**	**β-amino alcohol**	**Polyoxazoline**	**R^1^**	**R^2^**	**Reaction time (h)**	**Yield^a^** **(%)**

1	DFA	(*R*)-2-amino-1-butanol	**1**	H	Et	23	94^b^
2	DDFA	(*R*)-2-amino-1-butanol	**1**	Me	Et	18	93^b^
3	DFA	L-phenylalaninol	**2**	H	Bn	23	91^b^
4	DDFA	L-phenylalaninol	**2**	Me	Bn	18	90^b^
5	Diglycilic acid	(*R*)-2-amino-1-butanol	**3**	H	Et	23	98^b^
6	Dimethyl diglycilate	(*R*)-2-amino-1-butanol	**3**	Me	Et	18	96^b^
7	Triglycine	(*R*)-2-amino-1-butanol	**4**	H	Et	33	98^c^
8	Triethyl triglycinate	(*R*)-2-amino-1-butanol	**4**	Et	Et	30	96^d^
9	BTA	(*R*)-2-amino-1-butanol	**5**	H	Et	33	91^c^
10	TBTA	(*R*)-2-amino-1-butanol	**5**	Me	Et	30	93^d^
11	EDTA	(*R*)-2-amino-1-butanol	**6**	H	Et	28	95^e^
12	EGTA	(*S*)-2-amino-1-butanol	**7**	H	Et	28	93^e^

^a^Reaction conditions: *n*[R(CO_2_R^1^)*_x_*]:*n*(β-amino alcohol) = 1:1.0*x*–1:1.1*x* (molar ratio). ^b^Refluxed. ^c^Refluxed for 24 h and then stirred for 9 h at 125 °C after toluene removal. ^d^Refluxed for 24 h and then stirred for 6 h at 125 °C after toluene removal. ^e^Refluxed for 20 h and then stirred for 8 h at 135 °C.

### Enantioselective Rh(I)-catalyzed hydrosilylation of aromatic ketones with various polyoxazoline ligands

Enantiomerically pure chiral alcohols are key intermediates in the synthesis of numerous biologically active molecules [22]. For this reason, much effort has been made over the last 30 years to develop efficient techniques for asymmetric reduction of prochiral ketones. In particular, asymmetric catalysis provides organic chemists with a unique tool for their efficient synthesis [23], although none of these are, as yet, optimal [24–26]. In recent years, metal-catalyzed hydrosilylation of ketones has been investigated using chiral ligands [27–33], while enantioselective hydrogenation of prochiral ketones to optically active secondary alcohols is among the most fundamental subjects in modern synthetic chemistry. In this article, with the new polyoxazoline ligands in hand, the Rh-catalyzed hydrosilylation of aromatic ketones was explored (Table 2 and Table 3).

The reduction of acetophenone was first examined, since this is often used as the standard ketone in investigations of asymmetric hydrosilylation. The reaction of acetophenone with diphenylsilane in the presence of polyoxazolines **1**–**7** and [Rh(COD)Cl]_2_ was studied (Table 2, entries 1–15). First, the effect of temperature on the catalytic reaction with bisoxazoline **1** as a ligand was examined with THF as solvent (entries 1–3). At −10 °C, the reaction proceeded very slowly and only a 51% yield of 1-phenylethanol with low ee was obtained after 72 h (entry 1). When the temperature was raised to −5 °C, the acetophenone disappeared completely within 72 h to afford 1-phenylethanol in a yield of 86% with 89% ee (entry 2). As the temperature was raised to room temperature, the reaction was accelerated further, but the ee was slightly lower (entry 3). Therefore, the reaction temperature was optimized to −5 °C. As for solvent effect, the reaction displayed a preference for THF as solvent. The reaction proceeded very fast in CH_3_OH, but the ee was disappointingly low, only 55% (entry 4). However, 1-phenylethanol was obtained in high yield with high enantioselectivity in THF, exceeding that observed in either CCl_4_ or CH_3_OH (entries 2, 4 and 5). Catalyst concentrations were generally employed at 2 mol %, relative to acetophenone (entry 2), although loadings as low as 0.5 mol % could be used, a longer reaction time was required (entry 8). However, whereas a higher catalyst loading of 4 mol % worked well, there was no significant improvement in the ee (entry 9).

**Table 2:** Enantioselective Rh(I)-catalyzed hydrosilylation of acetophenone.

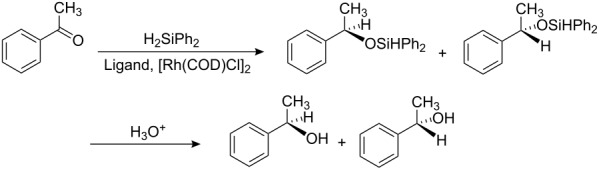								
**Entry**	**Ligand**	**Solvent**	**Time (h)**	***T*** **(°C)**	**Ligand/PhCOMe** **(mol %)**	**Yield^a^** **(%)**	**ee^b^** **(%)**	**Abs. config.^c^**

1	**1**	THF	72	−10	2.0	51	68	*R*
2	**1**	THF	72	−5	2.0	86	89	*R*
3	**1**	THF	48	rt	2.0	95	80	*R*
4	**1**	CH_3_OH	45	−5	2.0	81	55	*R*
5	**1**	CCl_4_	72	−5	2.0	78	70	*R*
6	**1**	THF	120	−5	2.0	87	90	*R*
7	**1**	THF	72	−5	1.0	81	81	*R*
8	**1**	THF	120	−5	0.5	76	73	*R*
9	**1**	THF	72	−5	4.0	86	90	*R*
10	**2**	THF	72	−5	2.0	84	97	*S*
11	**3**	THF	72	−5	2.0	64	32	*R*
12	**4**	THF	72	−5	2.0	74	37	*R*
13	**5**	THF	72	−5	2.0	70	20	*R*
14	**6**	THF	72	−5	2.0	78	55	*R*
15	**7**	THF	72	−5	2.0	74	48	*S*

^a^Conditions: [Rh(COD)Cl]_2_ (0.01 mmol), PhCOMe (2.0 mmol), Ph_2_SiH_2_ (3.2 mmol) and solvent (5.0 mL). ^b^The enantiomeric excess (ee) was determined by HPLC analysis using a Daicel Chiralcel OJ-H column. ^c^The absolute configurations were determined by optical rotation.

Under the optimized conditions, the reactions with **2**–**7** as ligands were carried out (Table 2, entries 10–15). The experimental results show that the ee with bisoxazoline **2** was higher than those with the bisoxazoline **3** and the polyoxazolines **4**–**7**, suggesting that the furan-containing bisoxazoline **2** held a good, rigid *C*_2_-symmetric chirality-inducing unit, which led to good enantioselectivity. By contrast, the benzene-containing trioxazoline **5** led to low enantioselectivity probably because its planar structure weakens the coordination of **5** to [Rh(COD)Cl]_2_. In addition, the experimental results also show that flexible linkers of oxazoline rings result in low enantioselectivities (entries 11, 12, 14, 15). Table 2 shows that bisoxazoline **2** with benzyl group (entry 10) gave a higher ee than its counterpart, bisoxazoline **1** with an ethyl group (entry 2): this result indicated that the bulkiness of the substituents on the oxazoline rings affected the enantioselectivities. As shown in Table 2, the absolute configurations of the resulting products were in good agreement with those of the corresponding chiral amino alcohols, because the enantioselectivities were determined solely by the chirality of oxazoline rings derived from the chiral amino alcohols.

The reduction of various prochiral aryl-substituted ketones was examined in order to evaluate the influence of ligand **2** on Rh(I)-catalyzed asymmetric hydrosilylation. Under the optimized conditions described above, the hydrosilylation of various aryl-substituted ketones catalyzed by [Rh(COD)Cl]_2_ in the presence of ligand **2** gave the corresponding secondary alcohols with good enantioselectivities. The results of all the reactions are shown in Table 3 in terms of the best ee values for each substrate. Reduction of acetophenone gave the corresponding alcohol in 84% yield and 97% ee (entry 1), while 2-acetylnaphthalene led to 85% yield of product with 94% ee (entry 2). It was found that *ortho*-substituted aromatic ketones resulted in lower enantioselectivities (entries 3, 4); a similar trend was observed for the substrate with a bulkier alkyl group at the carbonyl unit (entry 5). *para*-Substituted aromatic ketones were reduced to alcohols with high enantioselectivities, in most cases (entries 6–8).

**Table 3:** Asymmetric hydrosilylation of aromatic ketones catalyzed by ligand **2** and [Rh(COD)Cl]_2_ under optimized conditions.

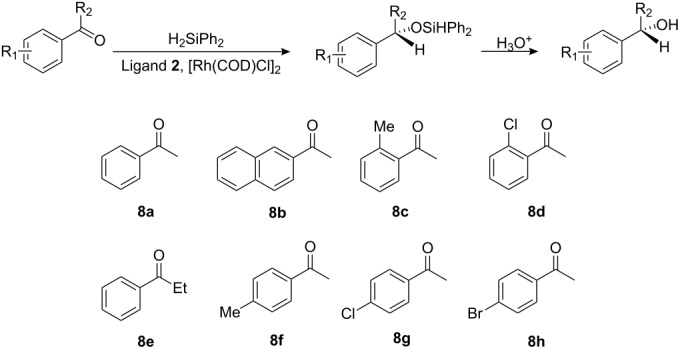				
**Entry**	**Ketone**	**Yield^a^** **(%)**	**ee^b^** **(%)**	**Abs. config.^c^**

1	**8a**	84	97	*S*
2	**8b**	85	94	*S*
3	**8c**	89	93	*S*
4	**8d**	71	90	*S*
5	**8e**	86	94	*S*
6	**8f**	81	96	*S*
7	**8g**	76	94	*S*
8	**8h**	79	96	*S*

^a^Conditions: ligand **2** (0.04 mmol), [Rh(COD)Cl]_2_ (0.01 mmol), ketone (2.0 mmol), Ph_2_SiH_2_ (3.2 mmol), THF (5.0 mL), −5 °C and 72 h. ^b^The enantiomeric excess (ee) was determined by HPLC analysis with chiral stationary phases. ^c^The absolute configurations were determined by optical rotation.

## Conclusion

Facile methods for the synthesis of polyoxazolines (**1**–**7**) are described, which are much simpler and more efficient in comparison to those reported in the literature. With these chiral ligands as templates, the rhodium-catalyzed asymmetric hydrosilylation of aromatic ketones was carried out. The effects of the linkers of oxazoline rings and the substituents on oxazoline rings of the ligands on the reaction were investigated, and compound **2** was identified as the best ligand of this family for the hydrosilylation of aromatic ketones. A study of the potential of this type of ligand for other metal-catalyzed asymmetric reactions is now in progress.

## Experimental

### General

Melting points were determined by the capillary method and are uncorrected. ^1^H NMR spectra were measured on a Varian Unity INOVI-500 NMR spectrometer or a Bruker Avance DPX300 NMR spectrometer, using TMS as internal standard. Infrared spectra were recorded on a Bruker Vector 22 FT-IR spectrometer. Mass spectra were taken on a LCQ DECA XP LC/MS system or a VG ZAB-HS mass spectrometer. Elemental analyses were carried out on a Perkin-Elmer 240C elemental analyzer. Optical rotation values were measured on a Polartronic HNQW 5 polarimeter. Enantiomeric excess (ee) was determined by HPLC analysis with chiral Daicel Chiralcel OJ-H (or OD-H, or OB-H) column on an Agilent HP-1100 HPLC instrument.

All solvents used for the synthesis were of analytical grade and were dried and freshly distilled under a nitrogen atmosphere prior to use. (*R*)- or (*S*)-2-amino-1-butanol and L-phenylalaninol were purchased from Fluka Chemical Co. 3,4-Dihydroxyfuran-2,5-dicarboxylic acid and its dimethyl ester were prepared as described in the literature [34]. Dimethyl diglycolate, triethyl aminotriacetate and trimethyl-1,3,5-benzenetricarboxylate were synthesized in our own laboratory. Other reagents were all of analytical grade.

### Syntheses of polyoxazoline ligands

#### 3,4-Dihydroxy-2,5-bis(4-substituted-oxazolin-2-yl)furan

Method A: 3,4-Dihydroxyfuran-2,5-dicarboxylic acid (0.75 g, 4.0 mmol), the chiral amino alcohol (8.8 mmol) and toluene (40 mL) were placed in a three-neck flask, fitted with a water segregator, a reflux condenser and a magnetic stirring bar. The mixture was refluxed and with continuous water removal for 23 h. Then, toluene was removed under reduced pressure. After cooling to ambient temperature, the residue was purified by silica gel column chromatography with ethanol as eluant to produce the desired compound.

Method B: 0.87 g (4.0 mmol) of dimethyl-3,4-dihydroxyfuran-2,5-dicarboxylate, 8.0 mmol of the chiral amino alcohol and 40 mL of toluene were added to a three-neck flask equipped with a water segregator, a reflux condenser and a magnetic stirring bar. The mixture was heated under reflux for 18 h with continuous removal of methanol and water. Then, the solvent was removed under reduced pressure. After cooling to room temperature, the residue was purified by column chromatography on silica gel with ethanol as eluant to yield the desired compound.

#### (−)-3,4-Dihydroxy-2,5-bis[4-(*R*)-ethyloxazolin-2-yl]furan (**1**)

This compound was obtained as a sticky colorless liquid in 94% yield by Method A, and in 93% yield following Method B. [α]_D_^20^ −10.0 (*c* 0.5, C_2_H_5_OH); IR (KBr): 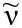 3276, 2968, 2941, 2880, 1652, 1518, 1055 cm^−1^; ^1^H NMR (500 MHz, CD_3_OD): δ 1.03 (t, *J* = 7.5 Hz, 6H), 1.62–1.73 (m, 4H), 3.10–3.15 (m, 2H), 3.57 (dd, *J* = 7.0, 11.5 Hz, 2H), 3.77 (dd, *J* = 3.5, 11.5 Hz, 2H), 4.55 (s, 2H) ppm; ESI-MS, *m*/*z* (%): 317 ([M+Na]^+^, 100); Anal. Calcd for C_14_H_18_N_2_O_5_: C, 57.13; H, 6.16; N, 9.52. Found: C, 57.32; H, 6.38; N, 9.40.

#### (+)-3,4-Dihydroxy-2,5-bis[4-(*S*)-benzyloxazolin-2-yl]furan (**2**)

This compound was obtained as a white solid in 91% yield by Method A, and in 90% yield following Method B. mp 162.0–163.5 °C; [α]_D_^20^ +18.0 (*c* 1.0, CH_3_OH); IR (KBr): 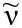 3344, 3034, 2935, 1605, 1496, 1454, 1378, 1322, 1057 cm^−1^; ^1^H NMR (500 MHz, CD_3_OD): δ 2.79 (dd, *J* = 7.0, 14.0 Hz, 2H), 2.88 (dd, *J* = 7.0, 13.5 Hz, 2H), 3.26–3.31 (m, 2H), 3.46 (dd, *J* = 6.5, 11.0 Hz, 2H), 3.62 (dd, *J* = 4.0, 11.0 Hz, 2H), 7.23–7.34 (m, 10H) ppm. Among the protons described above, two active hydrogens of OH were substituted by deuterium. ESI-MS, *m*/*z* (%): 419 ([M+H]^+^, 100); Anal. Calcd for C_24_H_22_N_2_O_5_: C, 68.89; H, 5.30; N, 6.69. Found: C, 68.51; H, 5.43; N, 6.86.

#### (−)-Bis{[4-(*R*)-ethyloxazolin-2-yl]methyl} ether (**3**)

Method A: Diglycolic acid (1.34 g, 10.0 mmol), (*R*)-2-amino-1-butanol (1.96 g, 22.0 mmol) and toluene (40 mL) were added to a three-neck flask with a water segregator, a reflux condenser and a magnetic stirring bar. The mixture was refluxed with continuous removal of water for 23 h. After cooling to room temperature, the solvent was removed under reduced pressure and the residue was purified by silica gel column chromatography with ethanol as eluant to give the pure title compound.

Method B: 1.62 g (10.0 mmol) of dimethyl diglycolate, 1.78 g (20.0 mmol) of (*R*)-2-amino-1-butanol and 40 mL of toluene were added to a three-neck flask equipped with a water segregator, a reflux condenser and a magnetic stirring bar. The mixture was refluxed for 18 h with continuous removal of methanol and water. After cooling to room temperature, the resulting mixture was concentrated and purified by silica gel column chromatography with ethanol as eluant to afford the pure desired compound.

This compound was obtained as a colorless oil in 98% yield by Method A, and in 96% yield following Method B. [α]_D_^20^ −27.0 (*c* 1.1, C_2_H_5_OH); IR (KBr): 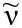 2970, 2886, 2578, 1588, 1464, 1410, 1308, 1128, 1056 cm^−1^; ^1^H NMR (500 MHz, CD_3_OD): δ 1.02 (t, *J* = 7.5 Hz, 6H), 1.63–1.70 (m, 4H), 3.09–3.50 (m, 2H), 3.58 (dd, *J* = 6.5, 12.0 Hz, 2H), 3.74 (dd, *J* = 3.5, 12.0 Hz, 2H), 3.94 (s, 4H) ppm; FAB-MS, *m*/*z* (%): 243 ([M+3]^+^, 5), 224 ([M−16]^+^, 10); Anal. Calcd for C_12_H_20_N_2_O_3_: C, 59.98; H, 8.39; N, 11.66. Found: C, 59.75; H, 8.51; N, 11.46.

#### (+)-*N*,*N*,*N*-Tris{[4-(*R*)-ethyloxazolin-2-yl]methyl}amine (**4**)

Method A: Aminotriacetic acid (0.77 g, 4.03 mmol), (*R*)-2-amino-1-butanol (1.19 g, 13.3 mmol) and toluene (40 mL) were added to a three-neck flask with a water segregator, a reflux condenser and a magnetic stirring bar. The mixture was refluxed for 24 h, then warmed to 125 °C and stirred for 9 h at the same temperature. After cooling to room temperature, the resulting mixture was chromatographed on silica gel using ethanol as eluant to yield the title compound.

Method B: Triethyl aminotriacetate (1.10 g, 4.0 mmol), (*R*)-2-amino-1-butanol (1.07 g, 12.0 mmol) and toluene (40 mL) were added to a three-neck flask with a water segregator, a reflux condenser and a magnetic stirring bar. The mixture was refluxed for 24 h, warmed to 125 °C and stirred for 6 h at the same temperature. After cooling to room temperature, the resulting mixture was chromatographed on silica gel using ethanol as eluant to obtain the title compound.

This compound was obtained as a white solid in 98% yield by Method A, and in 96% yield following Method B. [α]_D_^20^ +6.0 (*c* 1.0, CH_3_OH); mp 156.4–157.2 °C; IR (KBr): 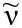 3385, 2970, 2946, 2886, 2567, 1634, 1404 cm^−1^; ^1^H NMR (500 MHz, CD_3_OD): δ 1.02 (t, *J* = 7.5 Hz, 9H), 1.64–1.69 (m, 6H), 3.11–3.13 (m, 3H), 3.56 (dd, *J* = 6.5, 11.5 Hz, 3H), 3.72 (s, 6H), 3.76 (dd, *J* = 3.5, 11.5 Hz, 3H) ppm; ESI-MS, *m*/*z* (%): 351 ([M+H]^+^, 100); Anal. Calcd for C_18_H_30_N_4_O_3_: C, 61.69; H, 8.63; N, 15.99. Found: C, 61.98; H, 8.80; N, 15.83.

#### (+)-1,3,5-Tris[4-(*R*)-ethyloxazolin-2-yl]benzene (**5**)

Method A: 1,3,5-Benzenetricarboxylic acid (0.84 g, 4.0 mmol), (*R*)-2-amino-1-butanol (1.18 g, 13.2 mmol) and toluene (40 mL) were added to a three-neck flask with a water segregator, a reflux condenser and a magnetic stirring bar. The mixture was refluxed for 24 h, the toluene was removed and the residue was stirred for 9 h at 125 °C. After cooling to room temperature, the resulting mixture was chromatographed on silica gel using ethanol as eluant to give the title compound.

Method B: Trimethyl-1,3,5-benzenetricarboxylate (1.00 g, 4.0 mmol), (*R*)-2-amino-1-butanol (1.07 g, 12.0 mmol) and toluene (40 mL) were placed in a three-neck flask, fitted with a water segregator, a reflux condenser and a magnetic stirring bar. The mixture was refluxed for 24 h, toluene was removed and the residue stirred for 6 h at 125 °C. After cooling to room temperature, the resulting mixture was chromatographed on silica gel using ethanol as eluant to afford the title compound.

This compound was obtained as a sticky orange liquid in 91% yield by Method A, and in 93% yield following Method B. [α]_D_^20^ +1.5 (*c* 1.0, CH_3_OH); IR (KBr): 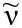 2971, 2941, 2886, 1611, 1558, 1464, 1429, 1358, 1064 cm^−1^; ^1^H NMR (500 MHz, CD_3_OD): δ 1.00 (t, *J* = 7.5 Hz, 9H), 1.61–1.69 (m, 6H), 3.08–3.12 (m, 3H), 3.56 (dd, *J* = 6.5, 11.5 Hz, 3H), 3.75 (dd, *J* = 4.0, 11.5 Hz, 3H), 8.70 (s, 3H) ppm; ESI-MS, *m*/*z* (%): 370 ([M+H]^+^, 100); Anal. Calcd for C_21_H_27_N_3_O_3_: C, 68.27; H, 7.37; N, 11.37. Found: C, 67.98; H, 7.63; N, 11.56.

#### (+)-*N*,*N*,*N*′,*N*′-Tetrakis{[4-(*R*)-ethyloxazolin-2-yl]methyl}ethylenediamine (**6**)

Ethylenediaminetetraacetic acid (1.17 g, 4.0 mmol), (*R*)-2-amino-1-butanol (1.57 g, 17.6 mmol) and toluene (40 mL) were added to a three-neck flask with a water segregator, a reflux condenser and a magnetic stirring bar. The mixture was refluxed for 20 h. Then toluene was removed and the residue stirred for 8 h at 135 °C. After cooling to room temperature, the resulting mixture was purified by silica gel column chromatography using ethanol as eluant to obtain 1.93 g of the title compound in 95% yield. Colorless oil; [α]_D_^20^ +198.0 (*c* 1.0, C_2_H_5_OH); IR (KBr): 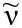 2966, 2875, 1590, 1062 cm^−1^; ^1^H NMR (500 MHz, CD_3_OD): δ 0.91–0.95 (m, 12H), 1.59–1.69 (m, 8H), 2.77 (t, *J* = 7.5 Hz, 4H), 3.06–3.11 (m, 4H), 3.28 (s, 8H), 3.51–3.58 (m, 8H) ppm; ESI-MS, *m*/*z* (%): 527 ([M+Na]^+^, 100); Anal. Calcd for C_26_H_44_N_6_O_4_: C, 61.88; H, 8.79; N, 16.65. Found: C, 62.10; H, 8.94; N, 16.86.

#### (−)-*N*,*N*,*N*′,*N*′-Tetrakis{[4-(*S*)-ethyloxazolin-2-yl]methyl}ethylene glycol-bis(2-aminoethyl) ether (**7**)

Ethylene glycol-bis(2-aminoethylether)-*N*,*N*,*N*′,*N*′-tetraacetic acid (1.53 g, 4.02 mmol), (*S*)-2-amino-1-butanol (1.58 g, 17.7 mmol) and toluene (40 mL) were added to a three-neck flask with a water segregator, a reflux condenser and a magnetic stirring bar. The mixture was refluxed for 20 h. The toluene was removed and the residue stirred for 8 h at 135 °C. After cooling to room temperature, the resulting mixture was purified by silica gel column chromatography using ethanol as eluant to afford 2.21 g of the title compound in 93% yield. [α]_D_^20^ −21.6 (*c* 1.0, C_2_H_5_OH); IR (KBr): 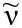 2968, 2938, 2880, 1580, 1066 cm^−1^; ^1^H NMR (500 MHz, CD_3_OD): δ 1.00 (t, *J* = 7.5 Hz, 12H), 1.51–1.64 (m, 8H), 2.94–2.99 (m, 4H), 3.22 (t, *J* = 2.5 Hz, 2H), 3.25 (t, *J* = 5.0 Hz, 2H), 3.29–3.31 (m, 2H), 3.49 (dd, *J* = 6.5, 11.5 Hz, 4H), 3.54 (s, 2H), 3.58 (s, 4H), 3.60–3.64 (m, 2H), 3.67 (s, 2H), 3.69 (dd, *J* = 3.5, 11.5 Hz, 4H), 3.76 (t, *J* = 5.5 Hz, 2H), 3.79 (t, *J* = 5.0 Hz, 2H) ppm; ESI-MS, *m*/*z* (%): 592 ([M−H]^−^, 100); Anal. Calcd for C_30_H_52_N_6_O_6_: C, 60.79; H, 8.84; N, 14.18. Found: C, 60.58; H, 8.98; N, 14.02.

### General procedure for the rhodium-catalyzed hydrosilylation of aromatic ketones

A mixture of polyoxazoline (0.04 mmol), [Rh(COD)Cl]_2_ (0.01 mmol) and aromatic ketone (2.0 mmol) in THF (5.0 mL) was stirred for 1 h at ambient temperature under a nitrogen atmosphere. After diphenylsilane (3.2 mmol) was added to the mixture at −5 °C, the reaction mixture was stirred at this temperature until the aromatic ketone was consumed. The reaction mixture was quenched with methanol (1.0 mL), then acidified with dilute hydrochloric acid at 0 °C and the organic layer was separated. The aqueous layer was extracted with diethyl ether or dichloromethane, and the organic layers were combined and dried with anhydrous Na_2_SO_4_. After purification by column chromatography on silica gel with CH_2_Cl_2_, the configuration of the product was determined by optical rotation and its enantiomeric excess was determined by HPLC analysis with chiral stationary phases.

## Supporting Information

Supporting information features spectroscopic data for the hydrosilylation products of aromatic ketones (**8a**–**8h**) and copies of ^1^H NMR and MS spectra for ligands (**1**–**7**).

File 1 Concise methods for the synthesis of chiral polyoxazolines and their application in asymmetric hydrosilylation.
