# Supplementary material for: Concise methods for the synthesis of chiral polyoxazolines and their application in asymmetric hydrosilylation
Source: Beilstein J Org Chem. 2010 Mar 25;6:29. doi: 10.3762/bjoc.6.29 (PMC2874315; doi:10.3762/bjoc.6.29)
Supplement: File 1 — Concise methods for the synthesis of chiral polyoxazolines and their application in asymmetric hydrosilylation. [file Beilstein_J_Org_Chem-06-29-s001.pdf]

Supporting Information

Concise methods for the synthesis of chiral polyoxazolines and their application in asymmetric hydrosilylation

Wei Jie Li*[1], Zun Le Xu[2] and Sheng Xiang Qiu[1]

Address: [1]Program for Natural Product Chemical Biology & Drug Discovery, South China Botanical Garden, Chinese Academy of Sciences, 723 Xingke Road, Tianhe District, Guangzhou 510650, China and [2]Department of Applied Chemistry, School of Chemistry and Chemical Engineering, Sun Yat-Sen University, 135 Xingang West Road, Haizhou District, Guangzhou 510275, China

Email: Wei Jie Li - weijieli1688@yahoo.com.cn

* Corresponding author

Data for the hydrosilylation products of aromatic ketones (**8a**–**8h**)

(*S*)-1-Phenylethanol: 84% yield; HPLC analysis (Daicel Chiralcel OJ-H, 5% *i*-PrOH/hexane, 0.5 mL/min; t_r (major) = 22.5, t_r (minor) = 25.4) gave the isomeric composition of the product: 97% ee; $[\alpha]_D^{20}$ −49.8 (*c* 1.0, CH_2Cl_2); ^1H NMR (300 MHz, $CDCl_3$): δ 1.35 (d, *J* = 6.79 Hz, 3H), 3.08 (br s, 1H), 4.71 (q, *J* = 6.04, 6.79 Hz, 1H), 7.15–7.23 (m, 5H) ppm; ESI-MS, *m*/*z* (%): 123 ($[M+H]^+$, 100); Anal. Calcd for $C_8H_{10}O$: C, 78.65; H, 8.25. Found: C, 78.32; H, 8.45.

(*R*)-1-Phenylethanol: 86% yield; HPLC analysis (Daicel Chiralcel OJ-H, 2% *i*-PrOH/hexane, 1.0 mL/min; t_r (major) = 10.6, t_r (minor) = 13.2) gave the isomeric composition of the product: 89% ee; $[\alpha]_D^{20}$ +43.6 (*c* 1.0, CH_2Cl_2); ^1H NMR (300 MHz, $CDCl_3$): δ 1.37 (d, *J* = 6.79 Hz, 3H), 3.05 (br s, 1H), 4.76 (q, *J* = 6.04, 6.79 Hz, 1H), 7.18–7.40 (m, 5H) ppm; ESI-MS, *m*/*z* (%): 123 ($[M+H]^+$, 100); Anal. Calcd for $C_8H_{10}O$: C, 78.65; H, 8.25. Found: C, 78.15; H, 8.52.

(*S*)-1-(2-Naphthyl)ethanol: 85% yield; HPLC analysis (Daicel Chiralcel OD-H, 2% *i*-PrOH/hexane, 1.0 mL/min; t_r (major) = 21.7, t_r (minor) = 23.6) gave the isomeric composition of the product: 94% ee; $[\alpha]_D^{20}$ −41.7 (*c* 1.0, $CHCl_3$); ^1H NMR (300 MHz, $CDCl_3$): δ 1.54 (d, *J* = 6.79 Hz, 3H), 2.16 (br s, 1H), 5.03 (q, *J* = 6.04, 6.79 Hz, 1H), 7.41–7.48 (m, 3H), 7.76–7.83 (m, 4H) ppm; ESI-MS, *m*/*z* (%): 173 ($[M+H]^+$, 100); Anal. Calcd for $C_{12}H_{12}O$: C, 83.69; H, 7.02. Found: C, 83.31; H, 7.24.

(*S*)-1-Phenylpropanol: 86% yield; HPLC analysis (Daicel Chiralcel OD-H, 4% *i*-PrOH/hexane, 0.5 mL/min; t_r (minor) = 17.4, t_r (major) = 20.4) gave the isomeric composition of the product: 94% ee; $[\alpha]_D^{20}$ −37.0 (*c* 0.50, $CHCl_3$); ^1H NMR (300

MHz, $CDCl_3$): δ 0.88 (t, *J* = 7.55 Hz, 3H), 1.65–1.81 (m, 2H), 1.96 (br s, 1H), 4.53 (t, *J* = 6.79 Hz, 1H), 7.21–7.30 (m, 5H) ppm; ESI-MS, *m*/*z* (%): 137 ($[M+H]^+$, 100); Anal. Calcd for $C_9H_{12}O$: C, 79.37; H, 8.88. Found: C, 79.12; H, 8.97.

(*S*)-1-(2-Methylphenyl)ethanol: 89% yield; HPLC analysis (Daicel Chiralcel OB-H, 3% *i*-PrOH/hexane, 0.5 mL/min; t_r (major) = 14.6, t_r (minor) = 22.2) gave the isomeric composition of the product: 93% ee; $[\alpha]_D^{20}$ −50.8 (*c* 0.05, C_2H_5OH); ^1H NMR (300 MHz, $CDCl_3$): δ 1.43 (d, *J* = 6.04 Hz, 3H), 1.85 (br s, 1H), 2.32 (s, 3H), 5.10 (q, *J* = 6.04, 6.79 Hz, 1H), 7.09–7.23 (m, 3H), 7.48 (d, *J* = 7.55 Hz, 1H) ppm; ESI-MS, *m*/*z* (%): 137 ($[M+H]^+$, 100); Anal. Calcd for $C_9H_{12}O$: C, 79.37; H, 8.88. Found: C, 79.05; H, 9.11.

(*S*)-1-(4-Methylphenyl)ethanol: 81% yield; HPLC analysis (Daicel Chiralcel OJ-H, 5% *i*-PrOH/hexane, 0.7 mL/min; t_r (major) = 10.9, t_r (minor) = 12.4) gave the isomeric composition of the product: 96% ee; $[\alpha]_D^{20}$ −54.1 (*c* 0.05, $CHCl_3$); ^1H NMR (300 MHz, $CDCl_3$): δ 1.45 (d, *J* = 6.79 Hz, 3H), 2.18 (br s, 1H), 2.36 (s, 3H), 4.82 (q, *J* = 6.04, 6.79 Hz, 1H), 7.15 (d, *J* = 8.30 Hz, 2H), 7.24 (d, *J* = 8.30 Hz, 2H) ppm; ESI-MS, *m*/*z* (%): 137 ($[M+H]^+$, 100); Anal. Calcd for $C_9H_{12}O$: C, 79.37; H, 8.88. Found: C, 79.21; H, 8.92.

(*S*)-1-(2-Chlorophenyl)ethanol: 71% yield; HPLC analysis (Daicel Chiralcel OD-H, 2% *i*-PrOH/hexane, 0.8 mL/min; t_r (minor) = 16.6, t_r (major) = 17.8) gave the isomeric composition of the product: 90% ee; $[\alpha]_D^{20}$ −57.2 (*c* 0.05, $CHCl_3$); ^1H NMR (300 MHz, $CDCl_3$): δ 1.38 (d, *J* = 6.79 Hz, 3H), 3.57 (br s, 1H), 5.18 (q, *J* = 6.04, 6.79 Hz), 7.10–7.25 (m, 3H), 7.49 (d, *J* = 7.55 Hz, 1H) ppm; ESI-MS, *m*/*z* (%): 157

([M+H]$^+$, 100); Anal. Calcd for C_8H_9ClO: C, 61.35; H, 5.79. Found: C, 61.14; H, 5.95.

(*S*)-1-(4-Chlorophenyl)ethanol: 76% yield; HPLC analysis (Daicel Chiralcel OJ-H, 5% *i*-PrOH/hexane, 0.5 mL/min; t_r (major) = 22.3, t_r (minor) = 26.0) gave the isomeric composition of the product: 94% ee; $[\alpha]_D^{20}$ −48.8 (*c* 0.06, $C_2H_5OC_2H_5$); ^{1}H NMR (300 MHz, $CDCl_3$): δ 1.44 (d, *J* = 6.79 Hz, 3H), 2.42 (br s, 1H), 4.81 (q, *J* = 6.04, 6.79 Hz, 1H), 7.25–7.28 (m, 4H) ppm; ESI-MS, *m*/*z* (%): 157 ([M+H]$^+$, 100); Anal. Calcd for C_8H_9ClO: C, 61.35; H, 5.79. Found: C, 61.23; H, 5.88.

(*S*)-1-(4-Bromophenyl)ethanol: 79% yield; HPLC analysis (Daicel Chiralcel OJ-H, 5% *i*-PrOH/hexane, 0.5 mL/min; t_r (major) = 22.2, t_r (minor) = 24.1) gave the isomeric composition of the product: 96% ee; $[\alpha]_D^{20}$ −37.6 (*c* 0.06, $CHCl_3$); ^{1}H NMR (300 MHz, $CDCl_3$): δ 1.38 (d, *J* = 6.04 Hz, 3H), 2.73 (br s, 1H), 4.77 (q, *J* = 6.04, 6.79 Hz, 1H), 7.15 (d, *J* = 8.30 Hz, 2H), 7.42 (d, *J* = 9.06 Hz, 2H) ppm; ESI-MS, *m*/*z* (%): 202 ([M+H]$^+$, 100); Anal. Calcd for C_8H_9BrO: C, 47.79; H, 4.51. Found: C, 47.35; H, 4.63.

Copies of ^{1}H NMR and MS spectra for ligands (1–7)

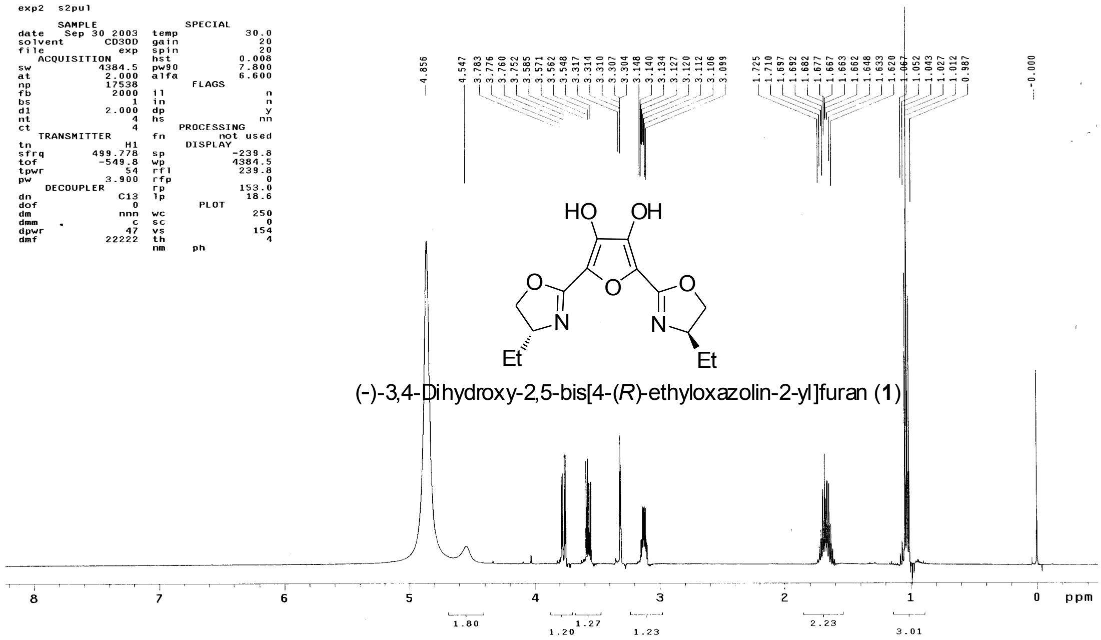

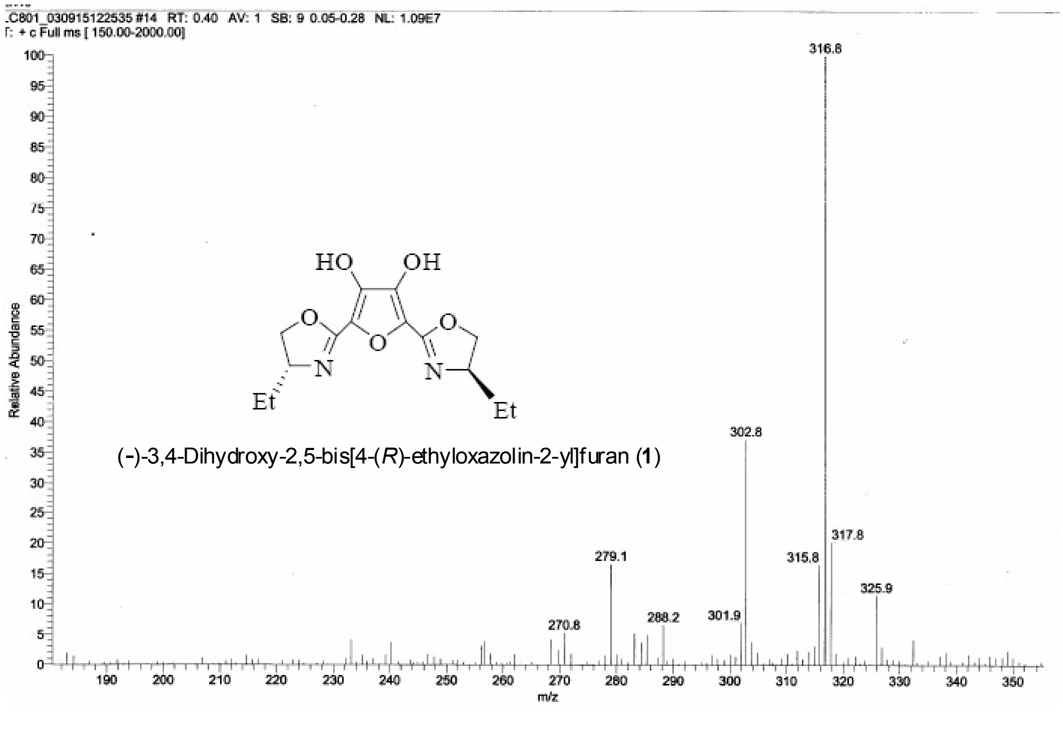

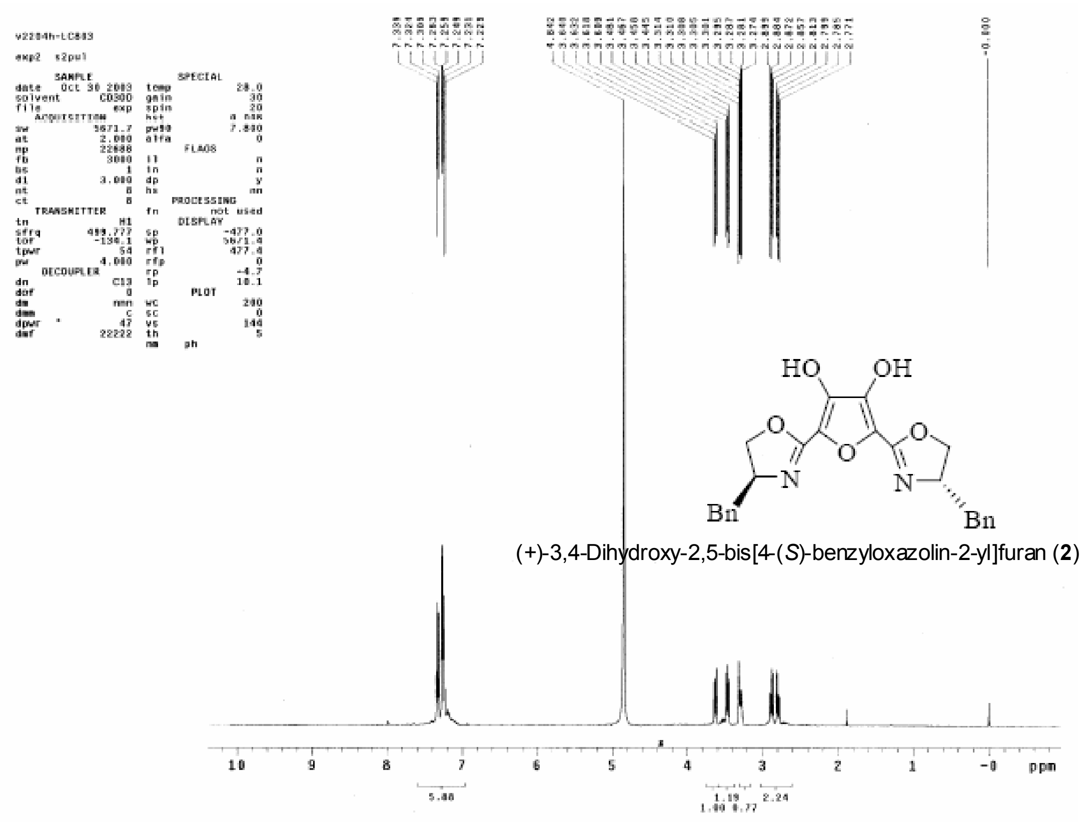

(+)-3,4-Dihydroxy-2,5-bis[4-(*S*)-benzyloxazolin-2-yl]furan (**2**)

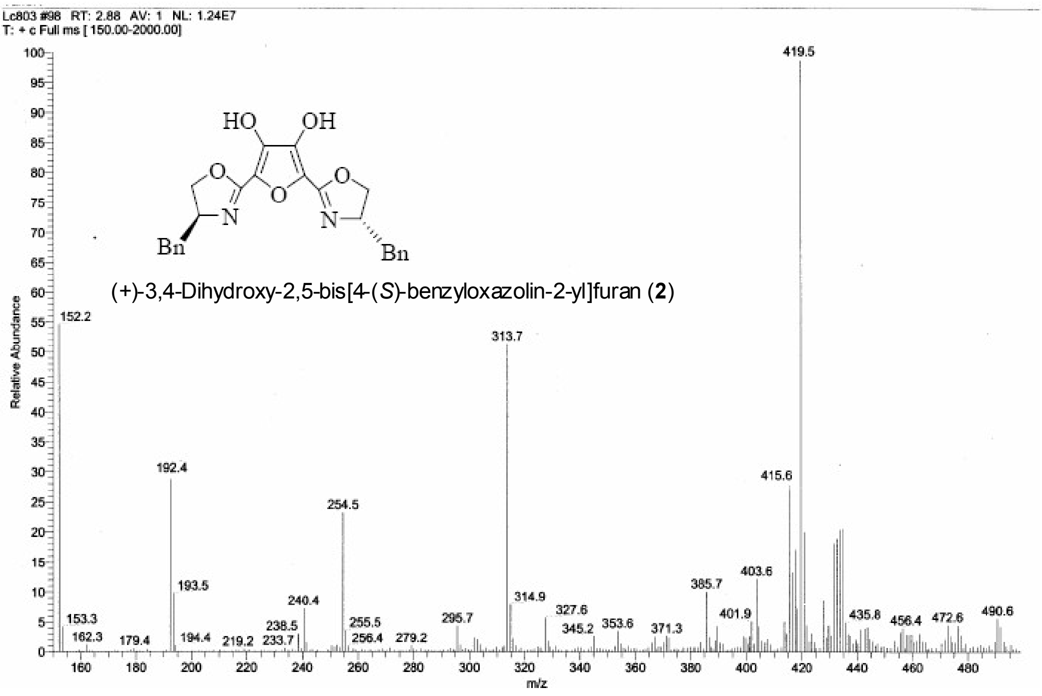

(+)-3,4-Dihydroxy-2,5-bis[4-(*S*)-benzyloxazolin-2-yl]furan (**2**)

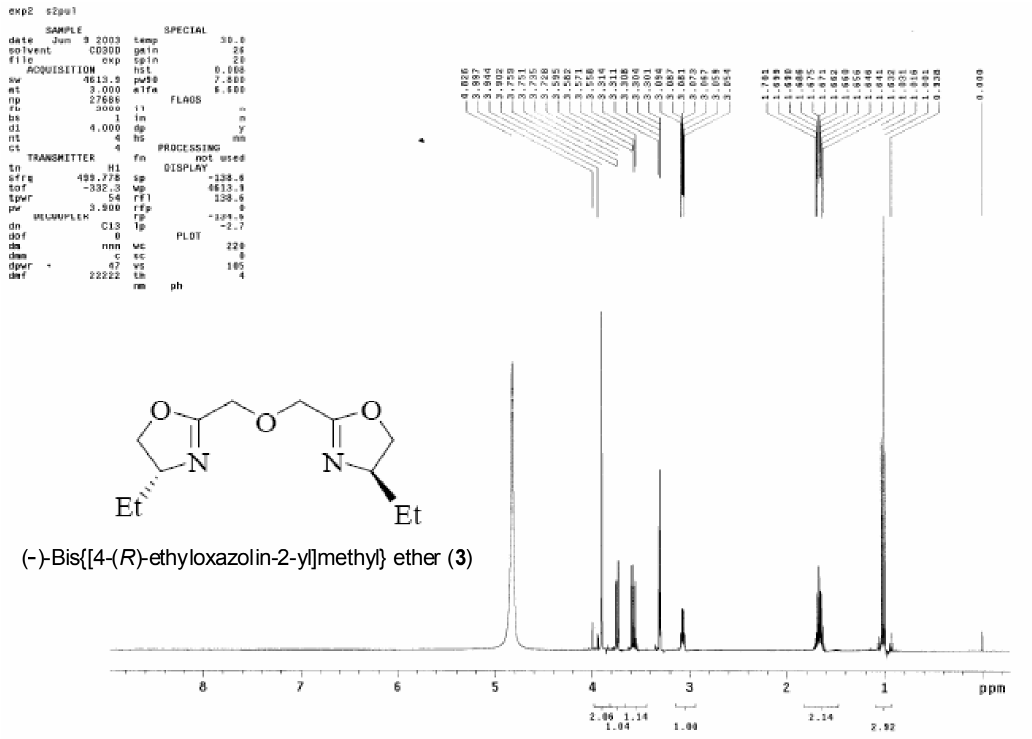
(-)-Bis{[4-(R)-ethyloxazolin-2-yl]methyl} ether (3)
ppm
2.06 1.14
1.04
1.00
2.14
2.92

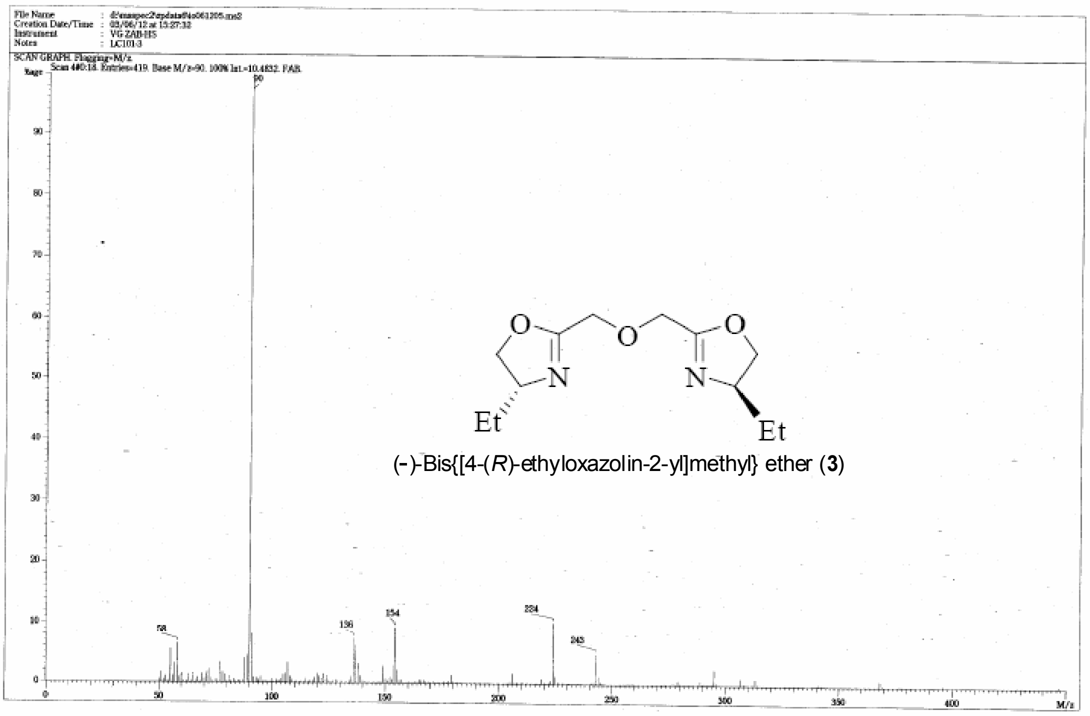
File Name
Creation Date/Time
Instrument
Notes
SCAN GRAPH. Flagging=M/z.
(-)-Bis{[4-(R)-ethyloxazolin-2-yl]methyl} ether (3)
58
136
154
224
243
M/z

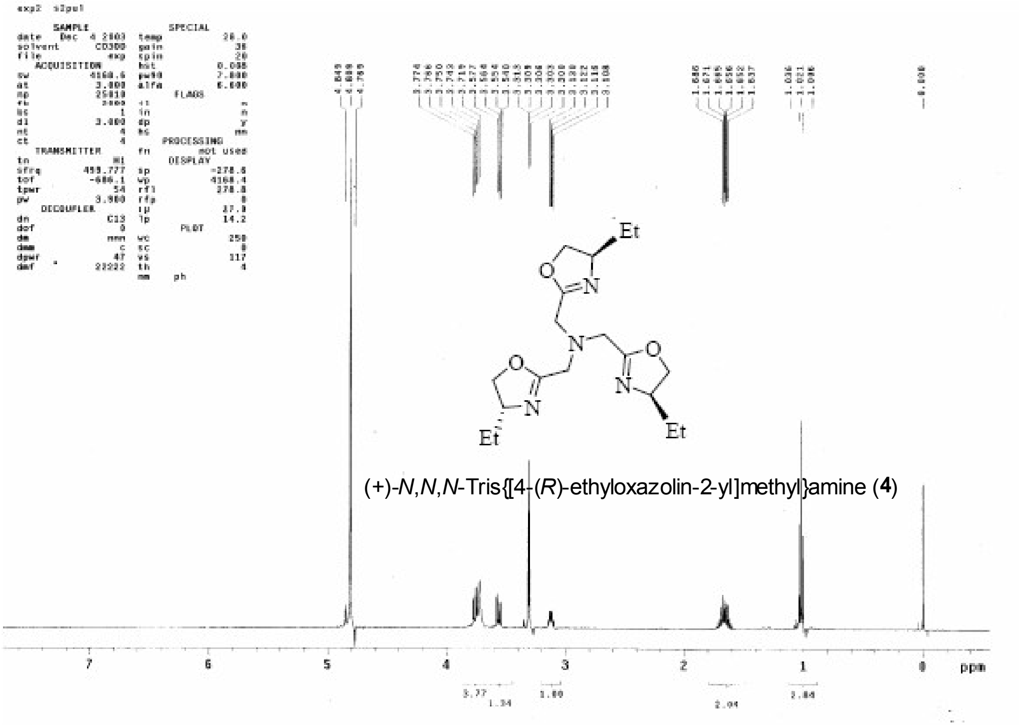

(+)-*N*,*N*,*N*-Tris{[4-(*R*)-ethyloxazolin-2-yl]methyl}amine (**4**)

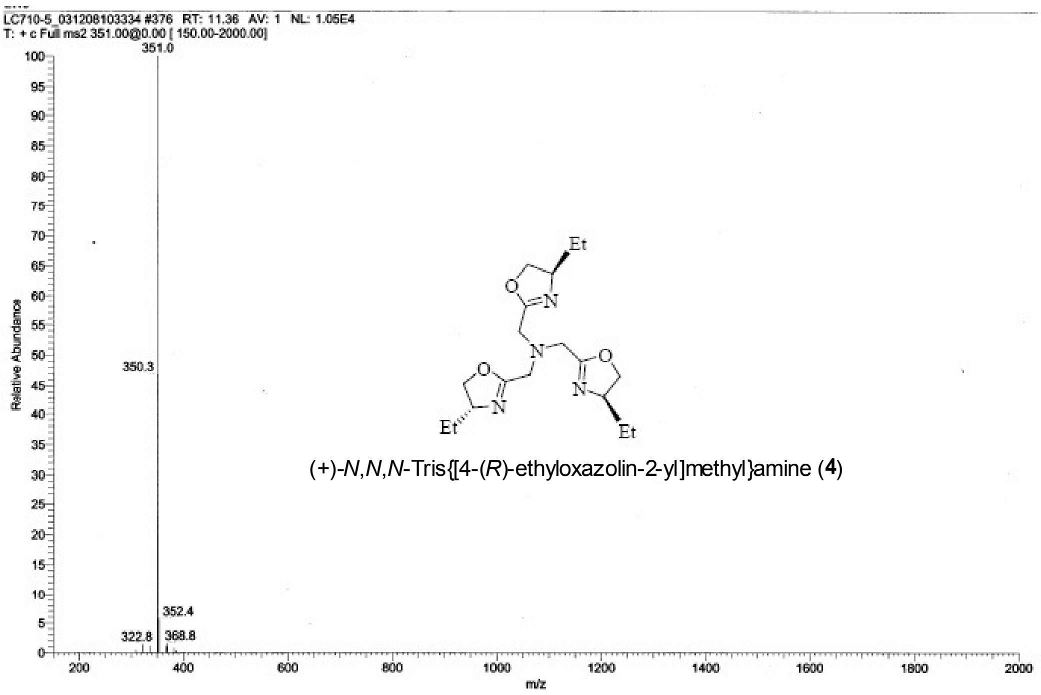

(+)-*N*,*N*,*N*-Tris{[4-(*R*)-ethyloxazolin-2-yl]methyl}amine (**4**)

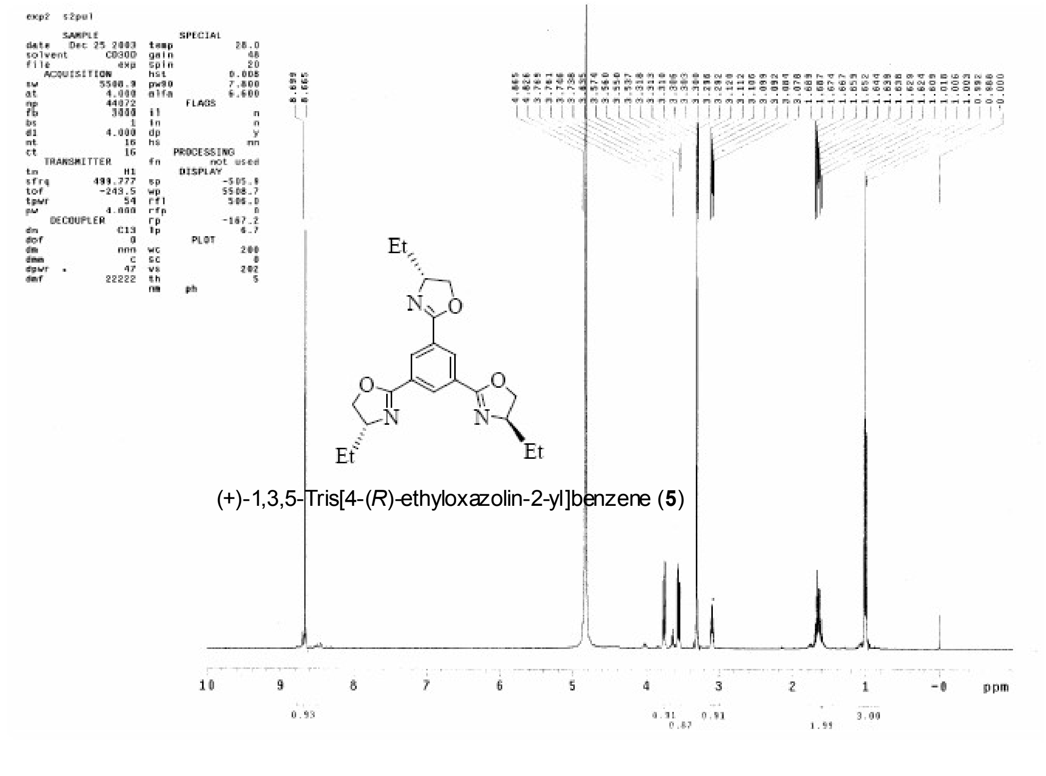

(+)-1,3,5-Tris[4-(*R*)-ethyloxazolin-2-yl]benzene (**5**)

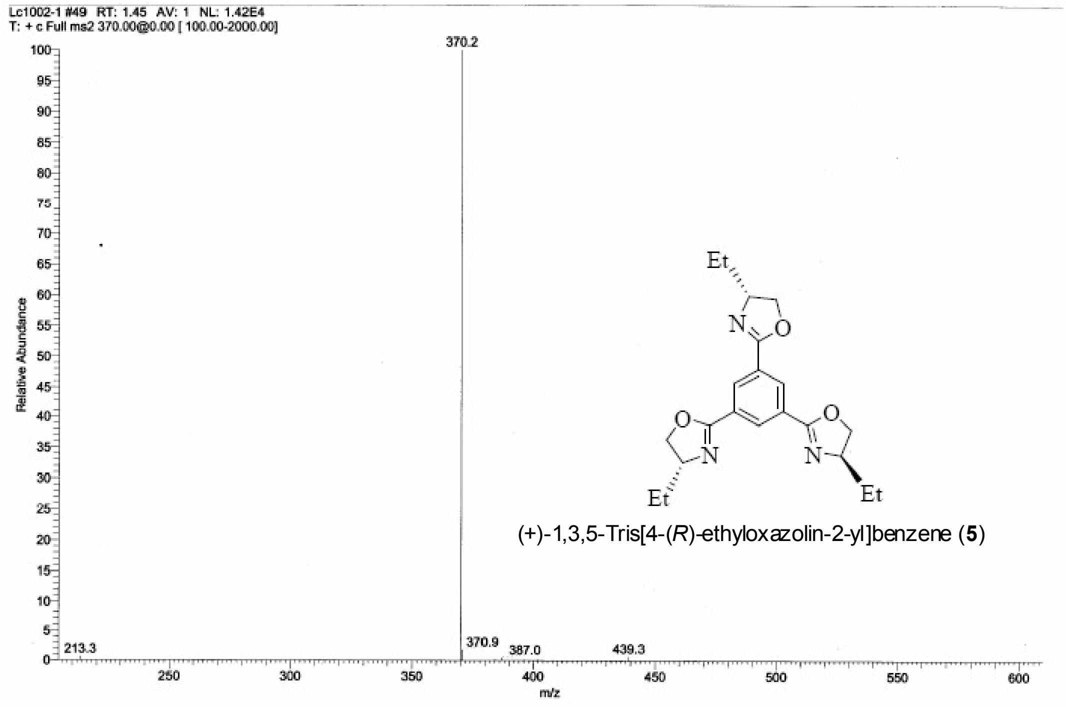

(+)-1,3,5-Tris[4-(*R*)-ethyloxazolin-2-yl]benzene (**5**)

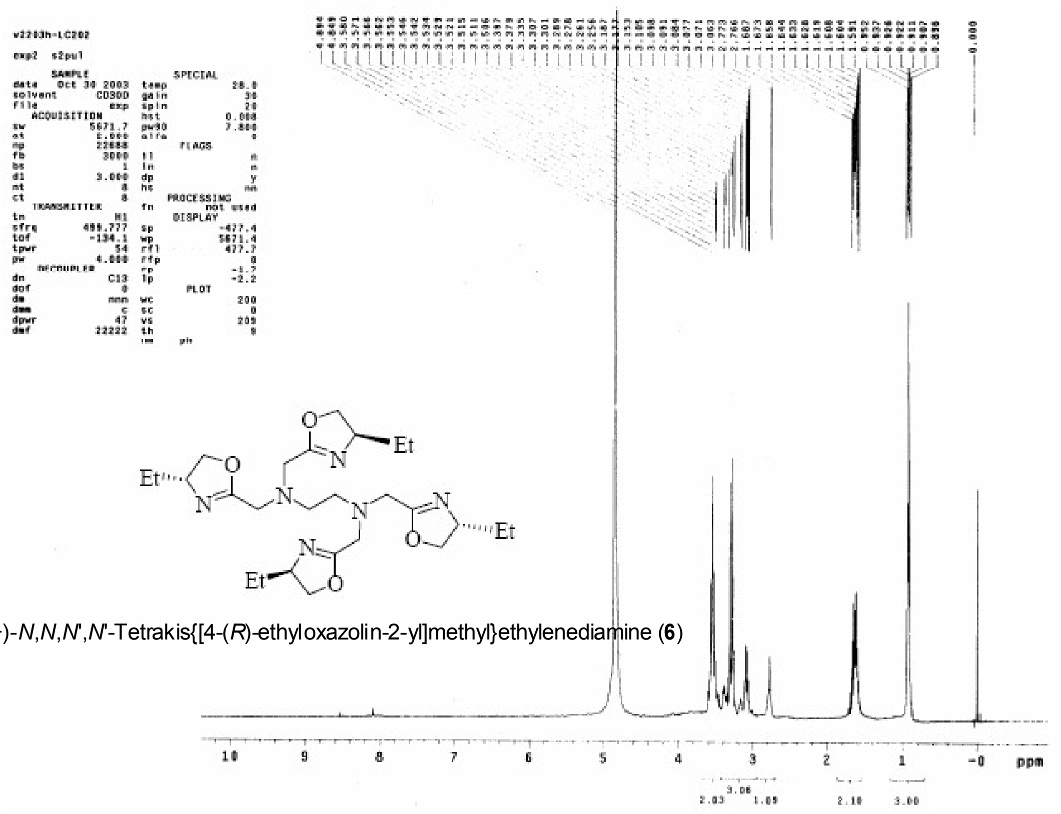

(+)-*N*,*N*,*N'*,*N'*-Tetrakis{[4-(*R*)-ethyloxazolin-2-yl]methyl}ethylenediamine (**6**)

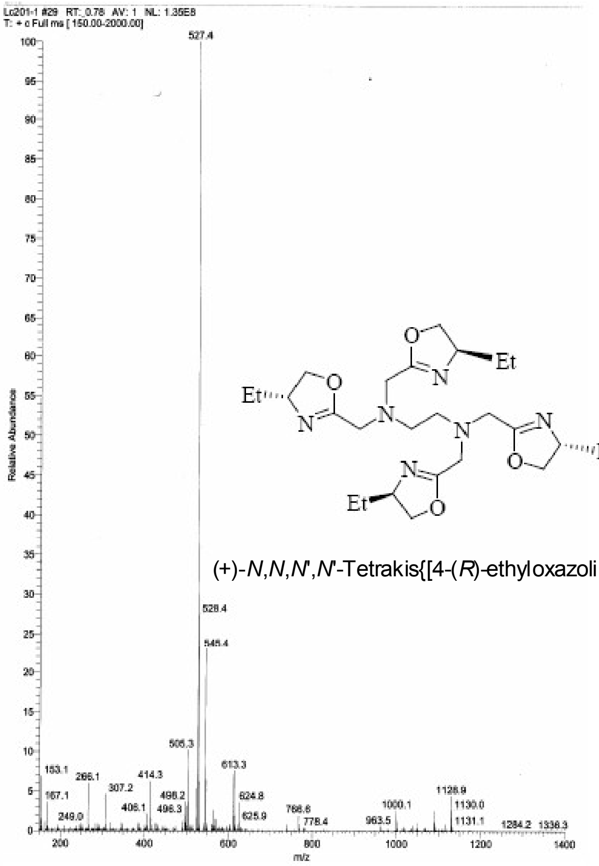

(+)-*N*,*N*,*N'*,*N'*-Tetrakis{[4-(*R*)-ethyloxazolin-2-yl]methyl}ethylenediamine (**6**)

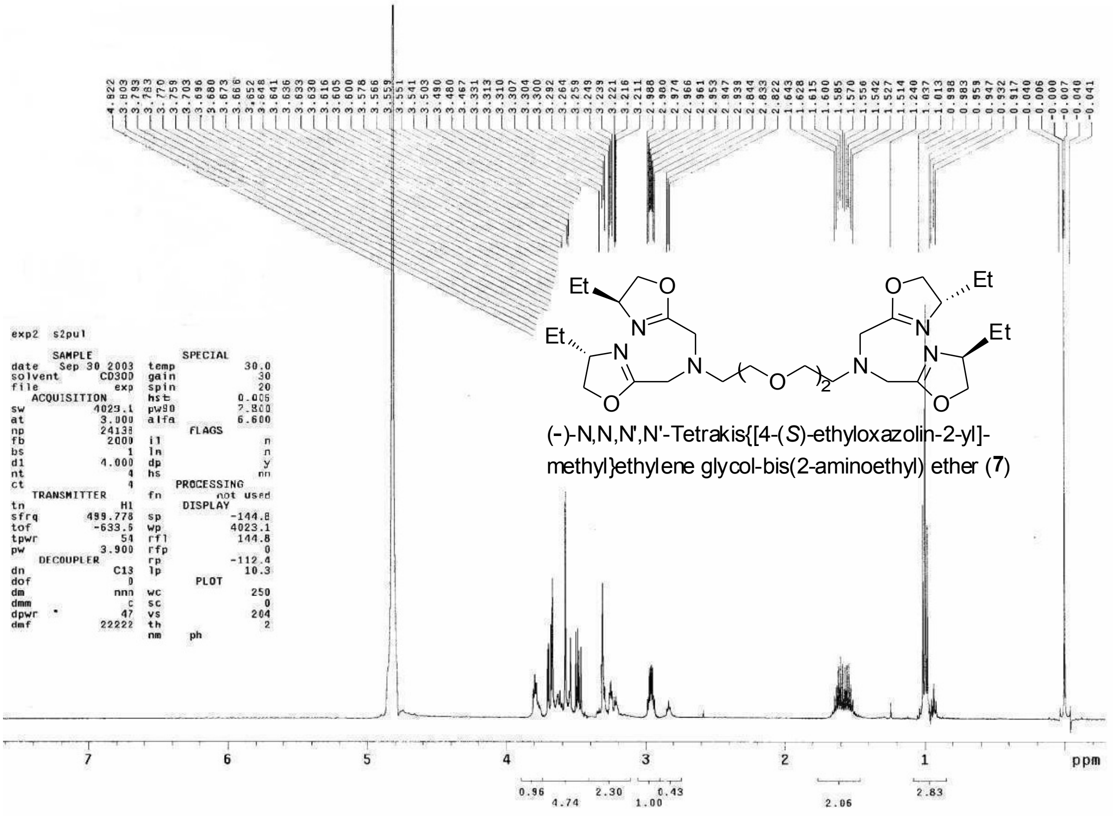
(-)-N,N,N',N'-Tetrakis{[4-(S)-ethyloxazolin-2-yl]-methyl}ethylene glycol-bis(2-aminoethyl) ether (7)
exp2 s2pul
ppm

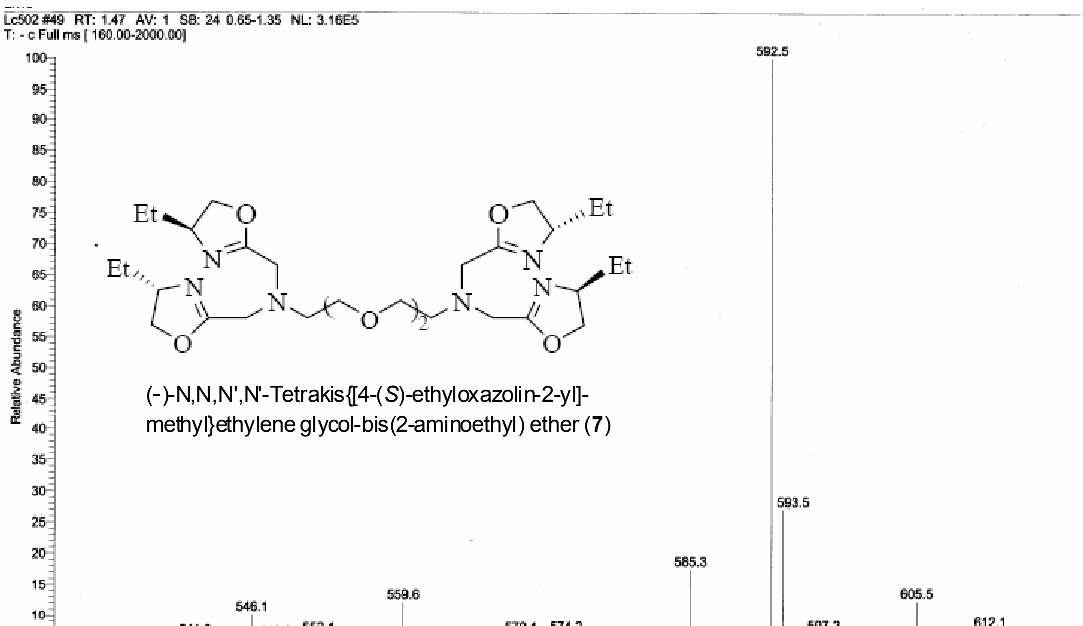
Lc502 #49 RT: 1.47 AV: 1 SB: 24 0.65-1.35 NL: 3.16E5
T: - c Full ms [160.00-2000.00]
(-)-N,N,N',N'-Tetrakis{[4-(S)-ethyloxazolin-2-yl]-methyl}ethylene glycol-bis(2-aminoethyl) ether (7)
Relative Abundance
m/z
592.5
593.5
585.3
605.5
612.1
559.6
546.1
